# Dried Leaf *Artemisia Annua* Improves Bioavailability of Artemisinin via Cytochrome P450 Inhibition and Enhances Artemisinin Efficacy Downstream

**DOI:** 10.3390/biom10020254

**Published:** 2020-02-07

**Authors:** Matthew R. Desrosiers, Alexis Mittleman, Pamela J. Weathers

**Affiliations:** 1 Department of Biology and Biotechnology, Worcester Polytechnic Institute, 100 Institute Road, Worcester, MA 01609, USA; mrdesrosiers@wpi.edu; 2 Department of Biomedical Engineering, Worcester Polytechnic Institute, 100 Institute Road, Worcester, MA 01609, USA; amittelman@wpi.edu

**Keywords:** *Artemisia annua*, artemisinin, cytochrome P450 inhibition, tissue distribution, inflammation

## Abstract

*Artemisia annua* L. and artemisinin, have been used for millennia to treat malaria. We used human liver microsomes (HLM) and rats to compare hepatic metabolism, tissue distribution, and inflammation attenuation by dried leaves of *A. annua* (DLA) and pure artemisinin. For HLM assays, extracts, teas, and phytochemicals from DLA were tested and IC_50_ values for CYP2B6 and CYP3A4 were measured. For tissue distribution studies, artemisinin or DLA was orally delivered to rats, tissues harvested at 1 h, and blood, urine and feces over 8 h; all were analyzed for artemisinin and deoxyartemisinin by GC-MS. For inflammation, rats received an intraperitoneal injection of water or lipopolysaccharide (LPS) and 70 mg/kg oral artemisinin as pure drug or DLA. Serum was collected over 8 h and analyzed by ELISA for TNF-α, IL-6, and IL-10. DLA-delivered artemisinin distributed to tissues in higher concentrations in vivo, but elimination remained mostly unchanged. This seemed to be due to inhibition of first-pass metabolism by DLA phytochemicals, as demonstrated by HLM assays of DLA extracts, teas and phytochemicals. DLA was more effective than artemisinin in males at attenuating proinflammatory cytokine production; the data were less conclusive in females. These results suggest that the oral consumption of artemisinin as DLA enhances the bioavailability and anti-inflammatory potency of artemisinin.

## 1. Introduction

In 2017, there were 219 million cases and 435,000 deaths from malaria according to the World Health Organization [1]. The frontline treatment is artemisinin combination therapy (ACT) that relies on semisynthetic derivatives of the drug artemisinin plus a codrug to eliminate patient parasites [2]. While effective, ACTs are often not financially feasible to those most in need in Africa, where 93% of malaria deaths occur [1]. Thus, there is great need for a more cost-effective alternative to ACTs that can treat malaria among the poorest of the world.

**Figure 1:**
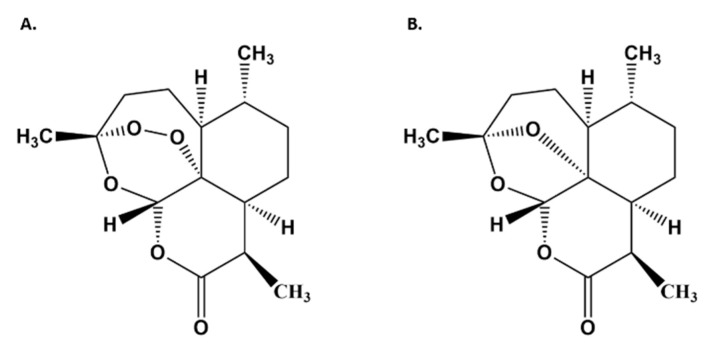
Chemical structures of artemisinin (**A**) and deoxyartemisinin (**B**).

The Chinese herb, *Artemisia annua* L., naturally produces and stores artemisinin (Figure 1A) in the glandular trichomes on its leaves, stems, and flowers [3]. Used for centuries as a tea infusion to treat a variety of illnesses including malaria [4], recent clinical data suggested that carefully prepared tea infusions made from the plant were as effective or better at eliminating malaria parasites as ACTs [5,6]. Furthermore, artemisinin delivered as a tea infusion or as dried leaves of *A. annua* (DLA) is significantly more bioavailable, crosses the intestine more efficiently, and has about four-fold greater solubility than pure artemisinin [7,8,9]. DLA also does not require extraction and purification, making it an affordable alternative to ACTs.

Artemisinin is metabolized by liver cytochrome P450s (CYPs), mainly CYP2B6 with a minor contribution from CYP3A4, to yield deoxyartemisinin (Figure 1B), crystal 7, deoxydihydroartemisinin, and 9,10 dihydrodeoxyartemisinin [10,11], all of which are therapeutically inactive. While the enhanced bioavailability of artemisinin afforded by DLA is partially attributed to increased solubility and intestinal transport [7,8], we posit that inhibition of CYP2B6 and/or CYP3A4 by DLA phytochemicals also plays a role in significantly increasing artemisinin bioavailability because more artemisinin would pass through the liver unmetabolized. In turn, that should also increase artemisinin distribution in tissues and organs with greater impact on biological responses, e.g., effects on inflammation. 

Despite significant bioavailability and pharmacokinetic differences between pure artemisinin and DLA-delivered artemisinin [9,12,13], to our knowledge, no studies have been done to determine absorption, distribution, metabolism and excretion (ADME) of the drug in vivo when delivered as DLA. There is only one report that determined tissue distribution of orally delivered pure artemisinin; it used a semiquantitative thin-layer chromatography (TLC) densitometric method to quantify artemisinin [14]. We are also not aware of any studies to determine in vivo gender differences in ADME of DLA-delivered artemisinin [15]. One study did show that the area under the curve (AUC) for pure artemisinin was two-fold higher in female rats compared to males after intraperitoneal administration. That same study showed artemisinin disappearance was 3.9-fold higher in liver microsomes from male vs. female livers, suggesting that artemisinin metabolism is gender dependent [15]. 

Artemisinin and other *A. annua* phytochemicals also have anti-inflammatory properties and are being investigated as therapeutics for several inflammatory diseases [16,17,18,19]. Artemisinin attenuates inflammation by blocking NF-κB and MAPK signaling pathways that lead to inflammatory cytokine production [16]. *A. annua* also produces other anti-inflammatory phytochemicals, including flavonoids and monoterpenes. The flavonoids, casticin and chrysosplenol-D, reduced in vitro and in vivo inflammation [20], and the monoterpene, 1,8-cineol (eucalyptol) inhibited the production of six inflammatory cytokines in vitro [21]. *A. annua* also produces rosmarinic and chlorogenic acids, each of which has anti-inflammatory activity [22]. With this rich mixture of anti-inflammatory phytochemicals, it was expected that *A. annua* could provide an alternative cost-effective therapeutic for inflammatory diseases. However, few in vivo studies have investigated whole plant *A. annua* treatment for inflammation.

The present study shows that artemisinin consumed orally as *A. annua* plant material (DLA) is more bioavailable from inhibition of the important artemisinin-metabolizing enzymes, CYP2B6 and CYP3A4, by compounds produced in the plant. Downstream of this first pass metabolism, DLA-delivered artemisinin is greater in tissues and organs and inflammation is decreased compared to pure artemisinin. We also show significant differences in gender with female rats absorbing higher amounts of artemisinin in several tissues regardless of delivery as DLA or pure drug. Together, these results better explain how *per os* DLA-delivered artemisinin bioavailability is greater than that from *per os* pure artemisinin. 

## 2. Materials and Methods 

### 2.1. Plant Material

We used dried leaves of the SAM cultivar of *Artemisia annua* L. (voucher MASS 317314) and *Artemisia afra* (SEN) (voucher LG0019529). SAM and SEN contained artemisinin between 1.0–1.2% and 0.02%, and flavonoid contents of 0.93–0.63% and 0.71% (*w*/*w*), respectively. *A. annua* was grown and processed as detailed in Weathers et al. 2014 [23]. *A. afra* was grown in Senegal and dried leaves were provided as a gift from Guy Mergaei, Université de Liège, Belgium. Artemisinin content was determined via GC-MS as detailed in Weathers and Towler 2012 [24]. Total flavonoid content was measured by the AlCl_3_ method of Arvouet-Grand et al. 1994 [25]. The *A. annua* used remains consistent in its phytochemical content over time as detailed in Weathers and Towler 2014 [26] and Gruessner et al. 2019 [27]. The *A. afra* used was from one batch of dried leaves for all experiments and was thus phytochemically consistent. Extensive phytochemical profiles of the *A. annua* and *A. afra* dried leaf materials used in this study are detailed in Weathers and Towler 2014 [26] and Munyangi et al. 2018 [28], respectively.

### 2.2. Chemicals and Reagents

Chemicals and reagents were from Sigma Aldrich (St. Louis, MO, USA) unless otherwise stated. Artemisinin was from Cayman Chemical (Ann Arbor, MI, USA); deoxyartemisinin from Toronto Research Chemicals (North York, ON, Canada). Lipopolysaccharide was *Escherichia coli* serotype O111:B4 and dissolved in sterile water at 1 mg/mL before storage at −20 °C. LEGEND MAX^TM^ Enzyme-linked immunosorbent assay (ELISA) kits for rat TNF-α and IL-6 were from BioLegend (San Diego, CA, USA). ELISA kits for rat IL-10 were from Thermo Fisher Scientific (Waltham, MA, USA). P450-Glo CYP2B6 and CYP3A4 kits were from Promega (Madison, WI, USA). Human liver microsomes (HLMs) from a 200-donor pool of male and female donors were from Sekisui XenoTech (Kansas City, KS, USA).

### 2.3. In Vitro P450 Inhibition

Promega P450-Glo kits for CYP2B6 or CYP3A4 were used with pooled HLMs (200 subjects) to determine an IC_50_ for each extract, tea, or phytochemical. Test concentrations ranged from 0.01–600 µM for each extract, tea, or phytochemical. *A. annua* extracts and teas were classified by their artemisinin content. For example, a 600 µM DLA extract is an extract with artemisinin at a 600 µM concentration. *A. afra* extracts and teas were made with a leaf dry weight equivalent to that used for *A. annua* extracts and teas such that a 600 µM *A. afra* extract was from the same leaf dry weight as a 600 µM extract of *A. annua*. Assays followed manufacturer instructions and plates were read on a luminescence plate reader. Methanol, acetonitrile, or acetone were cosolvent vehicles at ≤1% (v/v); a vehicle control was used for each comparison. IC_50_ values were determined through nonlinear regression using GraphPad Prism 7 (San Diego, CA, USA). All experiments had technical duplicates. Extracts, teas, and phytochemicals with an IC_50_ below 50 µM were repeated in biological triplicate.

### 2.4. Animals

Sprague-Dawley rats (Charles River Laboratories, Wilmington, MA, USA) between 200–300 g (age 5–8 weeks for males and 6–9 weeks for females) were used for all animal studies and housed at the Worcester Polytechnic Institute Vivarium on a 14 h light, 10 h dark cycle with ad libitum food and water until the day before an experiment. Food was withheld 14 h before experiments as animal chow has been shown to alter artemisinin bioavailability [12]. All animal work was approved by the Worcester Polytechnic Institute Institutional Animal Care and Use Committee (IACUC Protocol 15–69 and 18–105) and in accordance with the NIH Guide for the Care and Use of Laboratory Animals.

### 2.5. In Vivo Artemisinin Tissue Distribution

Artemisinin was delivered to rats *po* as either a slurry of powdered dried *A. annua* in water (DLA) or pure artemisinin in water with 12% DMSO. Artemisinin is essentially insoluble in water, so DMSO was used to enhance artemisinin solubility and achieve more consistent, replicable data. DLA-treated animals received 85 mg/kg dose of artemisinin in DLA slurry, the highest achievable dose that could be delivered while still conforming to animal welfare regulations. Initially we aimed to use the same dose of artemisinin in DLA and pure artemisinin-dosed animals, but preliminary experiments with a DLA-dosed rat and an artemisinin-dosed rat at 85 mg/kg showed no artemisinin was detectable in artemisinin treated animals. This was not surprising because pure artemisinin has very low oral bioavailability [29]; a previous study had to dose at 900 mg/kg of artemisinin to yield detectable artemisinin levels in the tissues [14]. Consequently, we increased the artemisinin dose in the control animals to 500 mg/kg and normalized amounts recovered in tissues, serum, urine, and feces to the delivered dose. After 1 h, animals were euthanized by CO_2_ inhalation; tissues and blood were harvested immediately after death confirmation. For longer experiments, rats housed in metabolic cages for the duration of the 8 h time course had urine, feces, and 0.4 mL blood samples collected 1, 2, 4 and 8 h after gavage to track artemisinin and one of its liver metabolites, deoxyartemisinin. Tissues were collected and flash frozen on liquid nitrogen 8 h after gavage. Blood was collected in heparin-free serum collection tubes, allowed to clot 20 min before centrifugation at 1,500 g for 10 min, and then stored at −80 °C until extraction.

### 2.6. Tissue Extraction 

Artemisinin and deoxyartemisinin were extracted from harvested tissues including heart, lungs, liver, spleen, kidneys, muscle, testes/ovaries, and brain. Briefly, a tissue sample was weighed, minced and then transferred to a Potter-Elvehjem tissue homogenizer. Water was added to yield a thick slurry and then homogenized. An aliquot was transferred into a new test tube and dichloromethane added at a 1:1 volume ratio. Samples were sonicated in a sonicating water bath for 30 min at ambient temperature and the organic layer decanted. The tissue residue was extracted twice again. Extracts were pooled, dried under nitrogen gas at ambient temperature, and stored at −20 °C until analysis. 

### 2.7. Serum and Urine Extractions 

Serum and urine were thawed before extraction, aliquot transferred into a new tube, and combined with dichloromethane at a 2:1 ratio. Each sample was vortexed, placed in the sonicating water bath for 30 min, the dichloromethane layer decanted, and extraction twice repeated. Extracts were pooled, dried under nitrogen, and stored at −20 °C until analysis.

### 2.8. Fecal Extractions 

Fecal samples collected at 1, 2, 4 and 8 h were stored at −80 °C until extraction. Before extraction, samples were vacuum dried for 72 h, ground with a mortar and pestle, wrapped in a tared Kimwipe, and weighed. Four milliliters dichloromethane was added to each sample in a test tube and then sonicated for 30 min and dried as described. Dried extracts were resuspended in 2 mL pentane and filtered through a 0.45 µm PVDF syringe filter into a new test tube to remove particulates. After a second extraction, pooled extracts were filtered and stored at −20 °C until analysis. 

### 2.9. Preparation of DLA Extracts and Teas

Briefly, 1 g DLA (*A. annua* or *A. afra*) was twice extracted with 10 mL methanol, sonicated and filtered as previously described. The process was repeated with the same plant material and the extract was dried under a mild stream of nitrogen before artemisinin content was analyzed by GC-MS. Tea at 5 g/L was prepared as described by Munyangi et al. (2018) [28].

### 2.10. Artemisinin and Deoxyartemisinin Analyses

Artemisinin and deoxyartemisinin were analyzed by gas chromatography mass spectrometry (GC-MS) as detailed previously [30]. Quantitation was based on standard curves from authenticated standards. 

### 2.11. Attenuation of Inflammation in Vivo

A rat model of systemic inflammation was modified from Bison et al. 2009 [31] with four test groups: Injection control, LPS control, LPS+AN, and LPS+DLA. Injection controls received a single intraperitoneal injection of sterile water as vehicle control for LPS. LPS controls received an intraperitoneal injection of LPS in water at 2.5 mg/kg to induce a systemic inflammatory response. LPS+AN, and LPS+DLA animals received a 2.5 mg/kg intraperitoneal LPS dose, followed immediately by oral gavage of pure artemisinin or DLA at a 70 mg/kg artemisinin dose, respectively; 70 mg/kg was the maximum artemisinin dose deliverable as DLA. Pure artemisinin was delivered with 12% DMSO to enhance solubility. Blood samples (0.4 mL) were taken via tail snip at 0 (taken immediately before injection), 1, 2, 4 and 8 h, clotted for 20 min at room temperature, and then centrifuged at 1500× *g* for 10 min to isolate serum. Serum was aliquoted and stored at −80 °C before ELISA analysis performed according to manufacturer.

### 2.12. Statistical Analysis

Statistical analysis was performed using GraphPad Prism 7. IC_50_ values were generated for CYP inhibition studies using nonlinear regression analysis in GraphPad Prism 7. Experiments were done in technical duplicate; three biological replicates were performed. IC_50_ values were compared to pure artemisinin by ANOVA. Each in vivo tissue distribution experiment compared the averages of three rats per group as permitted by the WPI IACUC. Students T-tests, used where appropriate, compared pure artemisinin to DLA dosed rats to determine statistical significance (*p* < 0.05). Each in vivo artemisinin elimination study compared the average of eight rats per group, as permitted by the WPI IACUC, and used students T-tests to compare DLA-dosed rats to rats dosed with pure artemisinin. Each in vivo inflammation experiment compared the averages of 5–6 rats per group except for the injection control in which there were three rats as was permitted by the WPI IACUC. ANOVA was used where appropriate to compare pure artemisinin- and DLA-dosed rats to LPS controls in inflammation studies.

## 3. Results

### 3.1. Inhibition of CYP2B6 and CYP3A4 by DLA

Extracts (DLA_me_ and A. afra_me_) and teas (DLA_tea_ and A. afra_tea_) of both *A. annua* and *A. afra* inhibited both CYP2B6 and CYP3A4 activity (Table 1). All extracts and teas had a lower IC_50_ than that for artemisinin alone, indicating that compounds other than artemisinin from DLA inhibited CYP2B6 and CYP3A4 activity. In addition to extracts and teas, some individually tested phytochemicals had inhibitory activity, but few were as strong as the whole extract (Table 1).

### 3.2. Artemisinin Delivered as DLA Increased Bioavailability in Some Tissues

**Figure 2:**
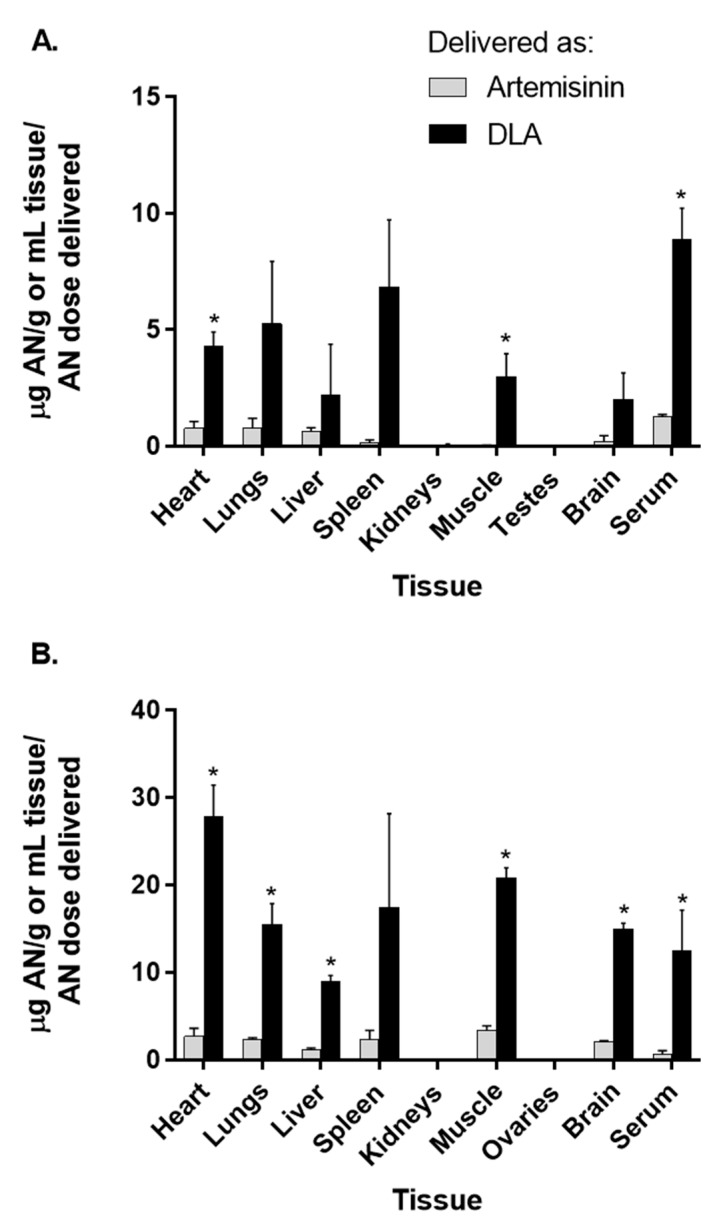
Artemisinin distribution in tissues and serum of male (**A**) and female (**B**) rats after oral delivery of artemisinin as dried leaf *A. annua* (DLA) or pure artemisinin. *n* = 3, *; *p* *≤* 0.05; error bars = SEM.

To determine how DLA alters distribution of artemisinin to tissues, we orally gavaged rats with DLA or pure artemisinin and measured artemisinin content. Compared to artemisinin-dosed males artemisinin in DLA-dosed rats at 1 h had significantly more artemisinin in the heart, muscle and serum (Figure 2A), while DLA-dosed females had significantly more artemisinin in the heart, lungs, liver, muscle, brain tissue, and serum (Figure 2B). In males, artemisinin accumulated mostly in serum followed by lungs, heart, liver, brain, spleen, muscle, and kidneys (Figure 2A). DLA-delivered artemisinin has greater bioavailability [7,8,12], so we expected more artemisinin in DLA-treated tissues; this was confirmed, as shown in Figure 2. 

**Figure 3:**
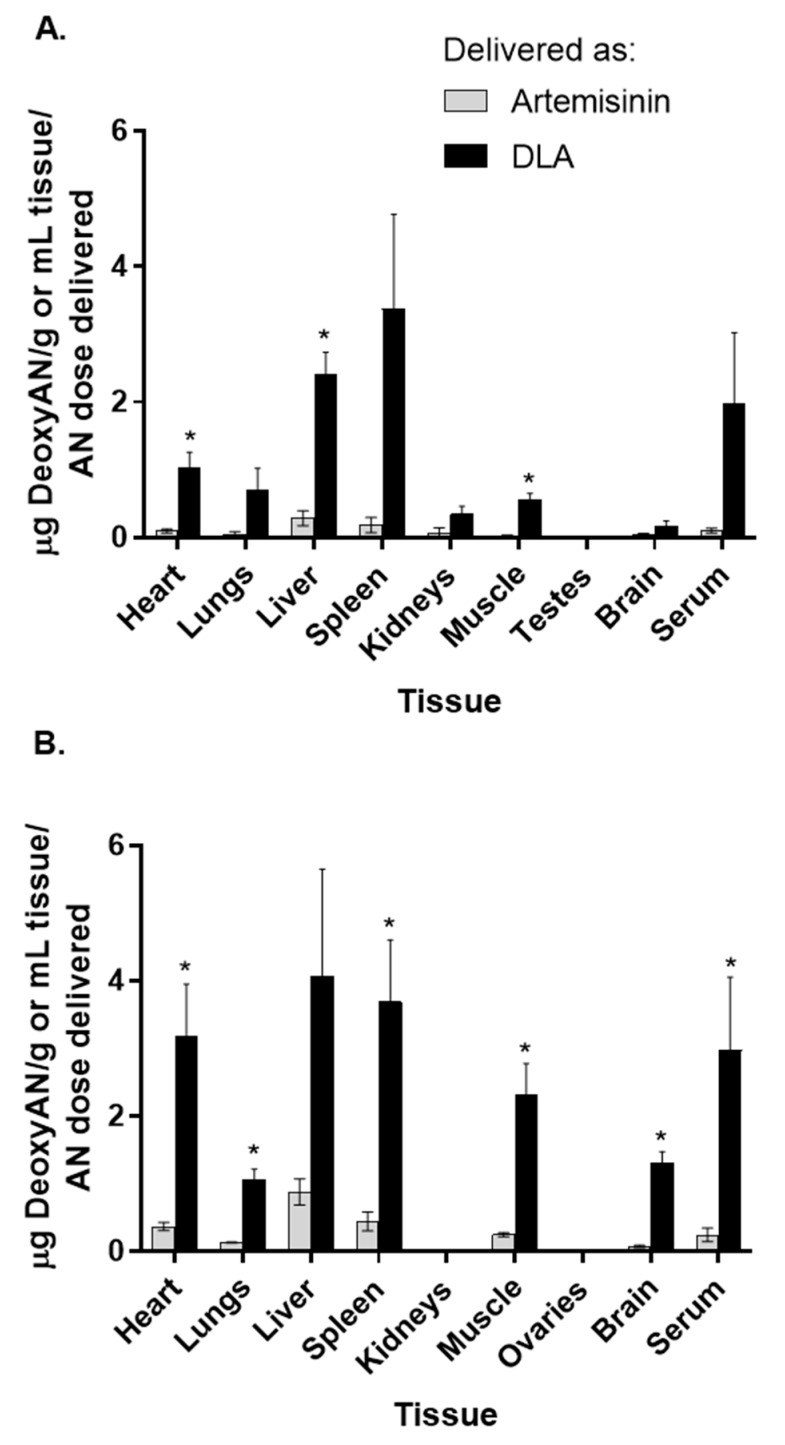
Deoxyartemisinin (deoxyAN) distribution in tissues and serum of male (**A**) and female (**B**) rats after oral delivery of artemisinin as dried leaf *A. annua* (DLA) or pure artemisinin. *n* = 3, *; *p* *≤* 0.05; error bars = SEM.

While artemisinin distributed well to most tissues, it cleared by 8 h. Neither artemisinin nor deoxyartemisinin was detectable in any tissues or serum 8 h postgavage. Serum was also collected at 2 and 4 h postgavage; however, neither artemisinin nor deoxyartemisinin was detectable in either gender. In male rats, heart, liver, and muscle had a significant difference in deoxyartemisinin distribution between DLA and pure artemisinin-treatments (Figure 3A). In female rats, however, there was a significant difference in deoxyartemisinin distribution to heart, lungs, spleen, muscle, brain, and serum (Figure 3B). DLA contains a small amount of deoxyartemisinin (7.5 mg/kg dose), so deoxyartemisinin detected in animals treated with DLA is likely not all from liver metabolism. Animals given pure artemisinin, however, did not receive any deoxyartemisinin, so any deoxyartemisinin detected in their tissues was due solely to artemisinin liver metabolism.

Distribution of artemisinin was significantly different in several tissues between males and females regardless of delivery mode (Table 2). At 1 h, females gavaged with pure artemisinin had more artemisinin in lungs, liver, muscle, and brain tissue than males (Table 2). Similarly, females gavaged with DLA had more artemisinin in the heart, lungs, liver, muscle, and brain tissue at 1 h compared to males (Table 2). In almost all tissues, although not always statistically significant, artemisinin and deoxyartemisinin were more abundant in females than males at 1 h. These data suggest females absorb and distribute artemisinin throughout the body more efficiently than males. There was, however, no difference in serum artemisinin levels at 1 h between female and male rats given either pure artemisinin or DLA (Table 2), suggesting that tissue distribution of artemisinin—but not absorption into the blood—is gender dependent.

### 3.3. Artemisinin Elimination through the Urine is Gender-Dependent

**Figure 4:**
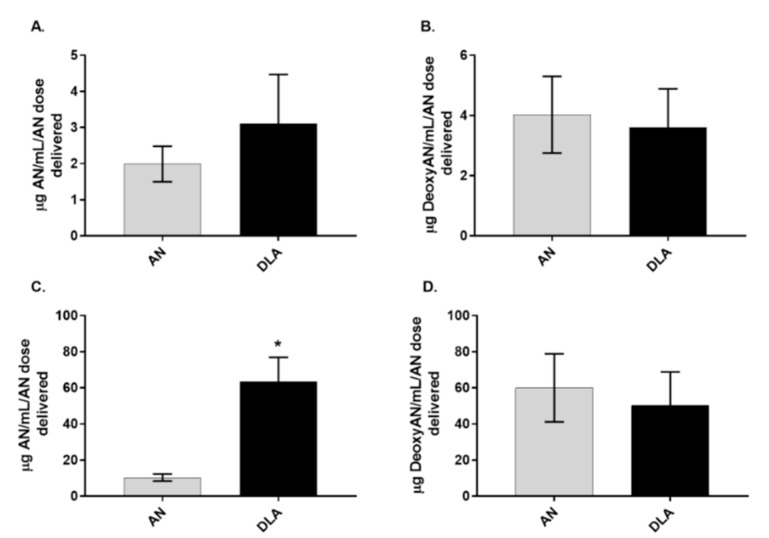
Artemisinin (AN) and deoxyartemisinin (DeoxyAN) accumulation in urine of male (**A**,**B**) and female (**C**,**D**) rats after oral delivery of artemisinin as dried leaf *A. annua* (DLA) or pure artemisinin. *n* = 8, *; *p* *≤* 0.05; error bars = SEM.

**Figure 5:**
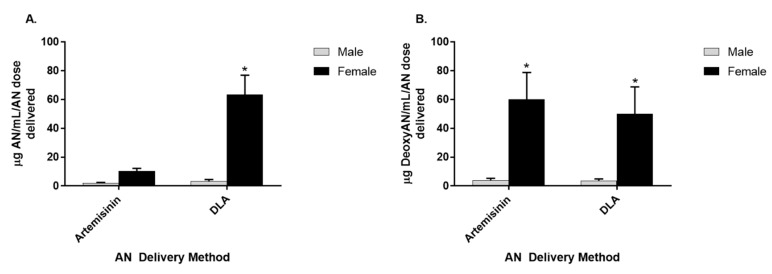
Artemisinin (**A**) and deoxyartemisinin (**B**) found in the urine of male and female rats orally gavaged with either pure artemisinin or dried leaves of *A. annua* (DLA). *n* = 8; * *p* *≤* 0.05; error bars = SEM.

To compare artemisinin elimination from DLA vs. pure artemisinin, urine collected over 8 h from female rats showed significantly more artemisinin elimination than males (Figure 4C). Because there is some deoxyartemisinin naturally present in DLA, those animals received 7.5 mg/kg of deoxyartemisinin in the DLA gavage. Animals treated with pure artemisinin did not receive any deoxyartemisinin. When comparing genders, females had more artemisinin in urine than males when given DLA. Females also had more deoxyartemisinin in urine than males, regardless of how artemisinin was delivered (Figure 5).

### 3.4. Artemisinin Elimination through Feces

Artemisinin and deoxyartemisinin was measured in the feces of rats up to 8 h after oral gavage with DLA or pure artemisinin (Supplementary Figure S1). As expected, most artemisinin and deoxyartemisinin was eliminated through feces, and was detected at later time points. Although total artemisinin and deoxyartemisinin was summed from the feces over 8 h, there were no significant differences in either artemisinin or deoxyartemisinin between DLA and pure artemisinin dosed animals (Supplementary Figure S2). We also measured fecal microbiome samples and found neither artemisinin nor DLA treatment affected the microbial population (Supplementary Table S1).

### 3.5. Attenuation of Inflammation by Artemisinin and DLA

**Figure 6:**
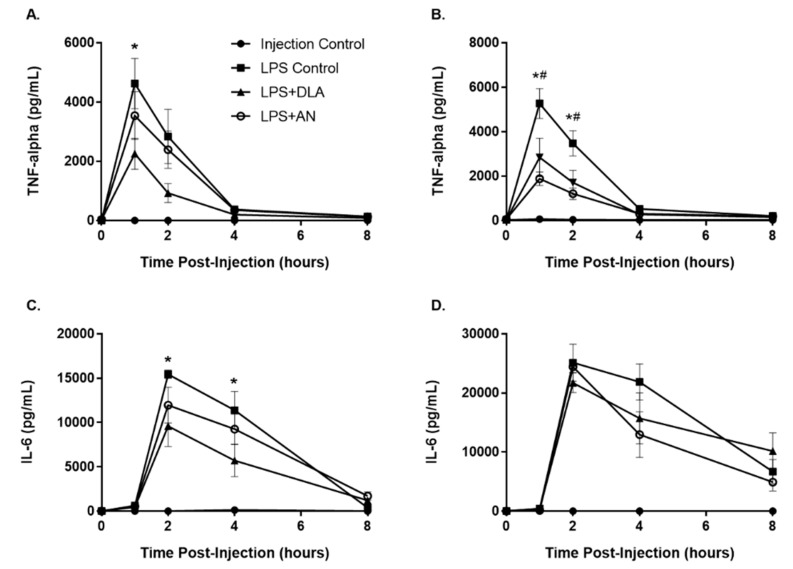
Production of proinflammatory cytokines TNF-α and IL-6 in male (**A**,**C**) and female (**B**,**D**) rats after LPS challenge and treatment with either pure artemisinin (AN) or DLA (equal artemisinin doses). *n* = 5–6 for experimental conditions, *n* = 3 for injection control; error bars = SEM; *, *p* *≤* 0.05 when comparing LPS+DLA to LPS Control; #, *p* *≤* 0.05 when comparing LPS+AN to LPS Control.

As expected, both male and female LPS controls responded to LPS challenge with a systemic inflammatory response. Serum TNF-α levels spiked quickly and peaked at about 4,600 pg/mL for males (Figure 6A) and 5,300 pg/mL for females (Figure 6B) at 1 hr after LPS injection. The spike in TNF-α was followed by a spike in serum IL-6 at 2 hours after LPS injection with males peaking at about 15,400 pg/mL (Figure 6C) and females peaking at about 25,100 pg/mL (Figure 6D). Interestingly, only DLA was effective at reducing serum TNF-α levels in males and only at the 1 h time point, while both DLA and pure artemisinin significantly reduced serum TNF-α levels at 1 and 2 h postinjection in females (Figure 6). Although artemisinin and DLA were effective at reducing TNF-α production in females, neither treatment had any effect on IL-6 serum levels in females. In males, however, DLA reduced serum IL-6 levels at 2 and 4 h postinjection. Overall, DLA was more effective at reducing inflammatory cytokine production in both genders, as expected based on the diversity of anti-inflammatory phytochemicals in DLA. 

We also measured the effects of DLA and pure artemisinin treatment on the anti-inflammatory cytokine IL-10. However, there were no significant differences in serum IL-10 levels, regardless of treatment (Figure S2). 

## 4. Discussion

*A*. *annua* produces many phytochemicals including kaempferol, chrysosplenetin, and quercetin [26,32] that have already been shown to inhibit either cytochrome P450 isoform 3A4 or 2B6, which are responsible for artemisinin metabolism in humans [10,33,34,35,36]. We hypothesized that the increased bioavailability of artemisinin afforded by DLA-delivery [9] is partly due to inhibition of hepatic metabolism by phytochemicals produced by DLA. The data presented in Section 3.1 confirm that phytochemicals present in DLA do inhibit liver P450s and this inhibition is part of the mechanism that leads to the previously reported increased bioavailability of DLA-delivered artemisinin [9]. Furthermore, since no individually tested phytochemicals produced stronger inhibition than the DLA extract, this inhibition is not solely due to any of the measured phytochemicals. It could, however, be from other as yet unidentified phytochemicals or a complex mixture of phytochemicals with moderate inhibitory activity. Future experiments will involve bioassay-guided fractionation of DLA extract to determine if there are any singular phytochemicals with potent inhibitory P450 activity.

In mice, artemisinin reaches peak serum concentration at 1 h postgavage [12]. Using TLC, Xinyi et al. (1985) showed orally delivered artemisinin accumulated mostly in the liver followed by brain, plasma, lung, kidney, muscle, heart, and spleen in male rats after 1 h [14]. However, it is unclear whether their TLC method could resolve artemisinin from deoxyartemisinin, an important liver metabolite. Here, using GC-MS analysis, we expanded on those results by determining differences when artemisinin was instead delivered as DLA and by comparing gender differences. DLA-delivered artemisinin distributed in higher quantities to many tissues in males and females. This was not surprising, given that DLA-delivered artemisinin was known to have increased bioavailability [9,12,13]. Interestingly, female rats had both better absorption of artemisinin into several tissues (Table 2) and higher amounts of artemisinin in their urine when given DLA (Figure 5). These data are consistent with a previous report showing females to have significantly decreased artemisinin metabolism compared to males in vitro [15].

The enhanced tissue distribution seen here and increased bioavailability afforded by DLA shown in the literature [9,12,13] led us to hypothesize DLA would be a more potent anti-inflammatory therapeutic than pure artemisinin. Indeed, the results shown in Figure 6 confirm this hypothesis. The enhanced efficacy against inflammatory cytokine production was likely due to increased bioavailability of artemisinin afforded by the inhibition of CYP2B6 and CYP3A4 by DLA phytochemicals. However, it is also plausible some DLA phytochemicals have their own inherent anti-inflammatory activity, and thus act additively with artemisinin to suppress inflammatory cytokine production. It is also possible that both mechanisms are in play. In either case, the benefit of the whole plant-based treatment is evident. While gender differences were expected, it was surprising that IL-6 production was affected neither by DLA nor pure artemisinin treatment in females, especially considering artemisinin is better absorbed and less efficiently metabolized in females compared to males [15].

We also posited that if DLA reduced inflammation, the reduction could occur by increasing anti-inflammatory cytokine production, e.g., IL-10. However, the results shown in Supplementary Figure S2 suggest that is unlikely. More likely is that DLA phytochemicals interact with molecular pathways responsible for inflammatory cytokine production, e.g., NF-κB and MAPK signaling, and should be further investigated in vitro with DLA extracts and individual phytochemicals.

Taken together, these results should be considered for potential drug–drug interactions with other medications taken by patients consuming *A. annua*. Consistent with the human pharmacokinetic study of Rath et al. (2004) [13], our results also indicate that delivery of artemisinin via *per os* consumption of *A. annua* can exceed the minimum antimalarial threshold of 9–10 µg/L blood [37] and should therefore obviate some concerns expressed by WHO (2012, 2019) [38,39] regarding inadequate delivery of artemisinin from *per os* consumption of *A. annua*. 

## 5. Conclusions

Although DLA extracts, teas, and phytochemicals inhibited CYP2B6 and CYP3A4, thereby showing that phytochemicals in DLA inhibit CYP450 enzymes that metabolize artemisinin, almost nothing is known about its tissue distribution and elimination. While the ADME of pure artemisinin was briefly explored previously by TLC [14], it was not compared to artemisinin delivered as DLA. Here, we showed that distribution of artemisinin to several tissues and serum significantly increased when delivered as DLA in male and female rats. Furthermore, the data suggest that artemisinin is differentially eliminated in males compared to females and differentially metabolized from DLA vs. pure artemisinin. More artemisinin therefore passes into the serum. Although we showed DLA is an effective anti-inflammatory agent in vivo, reducing TNF-α and IL-6 in males, in females, results were not as definitive because neither DLA nor pure artemisinin reduced IL-6 production, but did reduce TNF-α. Finally, neither artemisinin nor DLA affected IL-10 production, indicating that DLA and artemisinin likely reduce inflammation by inhibiting molecular signaling pathways responsible for proinflammatory cytokine production. Inhibition of the NF-κB and MAPK signaling cascade pathways by DLA extracts and phytochemicals require further investigation. Overall, these results enhance our understanding of how artemisinin delivered from *A. annua* is more bioavailable than when delivered as a pure drug.

## Figures and Tables

**Table 1:** Inhibition of CYP2B6 and CYP3A4 by DLA extracts, teas and phytochemicals.

Phytochemical or Extract	CYP2B6 IC_50_ (µM)	CYP3A4 IC_50_ (µM)
DLA_me_	6.07 **	4.93 ^#^
DLA_tea_	2.31 **	5.67 ^#^
A. afra_me_	7.89 **	5.18 ^#^
A. afra_tea_	11.27 **	5.59 ^#^
Arteannuin B	9.36 **	12.33 ^#^
Quercetin	19.86 *	4.59 ^#^
Artemisinin	27.75	>600
Chrysosplenol-D	52.06	22.96 ^#^
Chrysosplenetin	74.67	21.68 ^#^
Kaempferol	>600	33.84 ^#^
Deoxyartemisinin	110.86	>600
Camphor (+)	119.01	>600
Artemisinic Acid	122.83	239.5 ^#^
Camphor (-)	141.93	>600
Scopoletin	>600	410.19 ^#^
Chlorogenic Acid	>600	>600

* IC_50_ significantly lower than artemisinin; *p* ≤ 0.05; **p ≤ 0.0001; *n* = 3. ^#^ IC_50_ lower than artemisinin but no statistical difference determined because the artemisinin IC_50_ is higher than the highest tested concentration (600 µM).

**Table 2:** Gender-specific differences in artemisinin and deoxyartemisinin distribution to tissues in artemisinin- and DLA-treated rats.

	Artemisinin Treated				DLA Treated			
	Artemisinin (µg AN/g tissue/g AN delivered ± SD)		Deoxyartemisinin (µg DeoxyAN/g tissue/g AN delivered ± SD)		Artemisinin (µg AN/g tissue/g AN delivered ± SD)		Deoxyartemisinin (µg DeoxyAN/g tissue/g AN delivered ± SD)	
Tissue	Male	Female	Male	Female	Male	Female	Male	Female
**Heart**	0.77 ± 0.49	2.70 ± 1.87	0.097 ± 0.050	0.37 ± 0.12 *	4.30 ± 1.06	27.81 ± 7.25 *	1.02 ± 0.42	3.18 ± 1.54
**Lungs**	0.79 ± 0.69	2.37 ± 0.26 *	0.055 ± 0.054	0.13 ± 0.018 *	5.26 ± 4.64	15.56 ± 4.63 *	0.70 ± 0.65	1.06 ± 0.31
**Liver**	0.62 ± 0.30	1.23 ± 0.26 *	0.29 ± 0.19	0.88 ± 0.39	2.19 ± 3.79	8.96 ± 1.40 *	2.42 ± 0.55	4.06 ± 3.18
**Spleen**	0.14 ± 0.24	2.37 ± 2.05	0.19 ± 0.20	0.44 ± 0.28	6.82 ± 5.03	17.49 ± 21.37	3.38 ± 2.79	3.69 ± 1.83
**Kidneys**	0.00 ± 0.00	0.00 ± 0.00	0.074 ± 0.12	0.00 ± 0.00	0.048 ± 0.096	0.00 ± 0.00	0.35 ± 0.23	0.00 ± 0.00*
**Muscle**	0.019 ± 0.034	3.41 ± 0.85 *	0.025 ± 0.018	0.25 ± 0.049 *	2.99 ± 1.68	20.84 ± 2.26 *	0.57 ± 0.15	2.31 ± 0.93 *
**Testes/Ovaries**	0.00 ± 0.00	0.00 ± 0.00	0.00 ± 0.00	0.00 ± 0.00	0.00 ± 0.00	0.00 ± 0.00	0.00 ± 0.00	0.00 ± 0.00
**Brain**	0.22 ± 0.38	2.05 ± 0.35 *	0.038 ± 0.043	0.075 ± 0.034	2.02 ± 1.96	15.00 ± 1.31 *	0.18 ± 0.12	1.30 ± 0.36 *
**Serum**	1.29 ± 0.11	0.67 ± 0.75	0.10 ± 0.061	0.24 ± 0.20	8.90 ± 2.63	12.49 ± 9.34	1.97 ± 2.10	2.97 ± 2.17

AN, artemisinin; deoxyAN, deoxyartemisinin; SD, standard deviation; *n* = 3; *, *p* *≤* 0.05 when compared to opposite gender.

## Supplementary Materials

The following are available online at https://www.mdpi.com/2218-273X/10/2/254/s1, Figure S1: Artemisinin (AN) and deoxyartemisinin (DeoxyAN) accumulation in feces of male (**A**,**B**) and female (**C**,**D**) rats after oral delivery of artemisinin as dried leaf A. annua (DLA) or pure artemisinin. *n* = 7–8, *; *p* < 0.05; error bars = SEM., Figure S2: Production of anti-inflammatory cytokine IL-10 in male (**A**) and female (**B**) rats after LPS challenge and treatment with either pure artemisinin (AN) or DLA (equal artemisinin doses). *n* = 5–6 for experimental conditions, *n* = 3 for injection control; error bars = SEM, Table S1: Microbiome population by Phylum 1 h and 8 h after treatment with DLA or pure artemisinin in male rats.
