# Supplementary material for: Dried Leaf Artemisia Annua Improves Bioavailability of Artemisinin via Cytochrome P450 Inhibition and Enhances Artemisinin Efficacy Downstream
Source: Biomolecules. 2020 Feb 7;10(2):254. doi: 10.3390/biom10020254 (PMC7072484; doi:10.3390/biom10020254)
Supplement: Supplementary file 1 [file biomolecules-10-00254-s001.pdf]

Appendix A: Supplementary Material

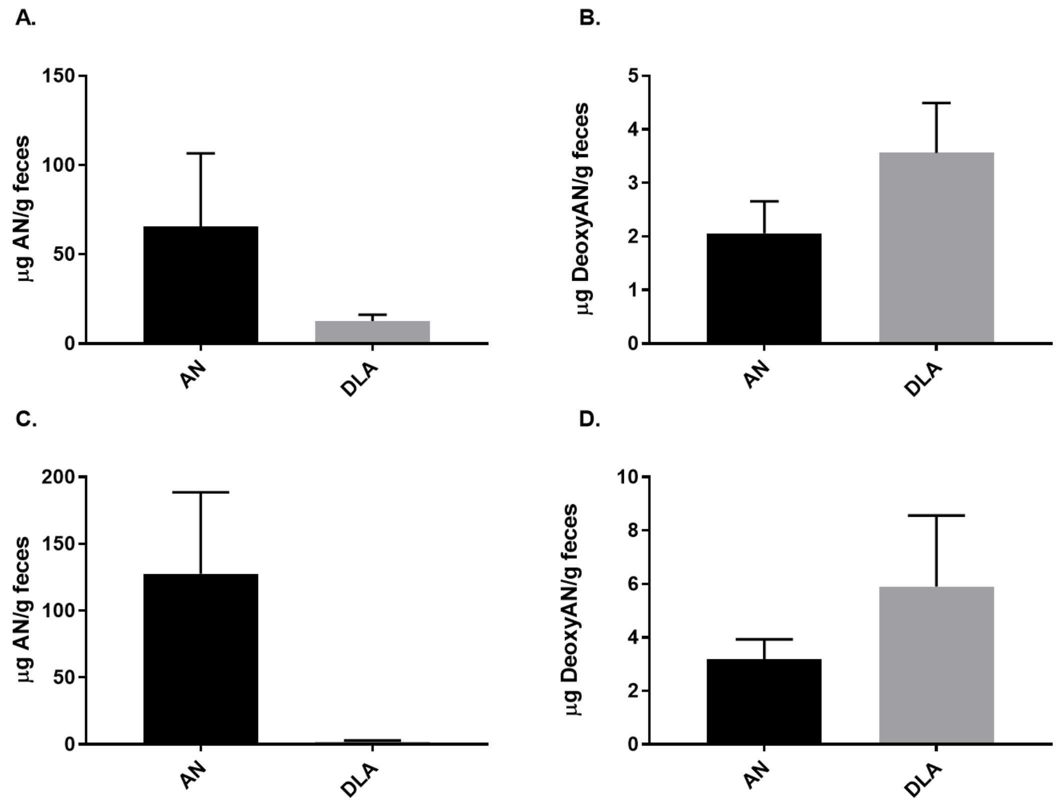

Figure A1. Artemisinin (AN) and deoxyartemisinin (DeoxyAN) accumulation in feces of male (**A**, **B**) and female (**C**, **D**) rats after oral delivery of artemisinin as dried leaf *A. annua* (DLA) or pure artemisinin. $n = 7–8$, *; $p < 0.05$; error bars = SEM.

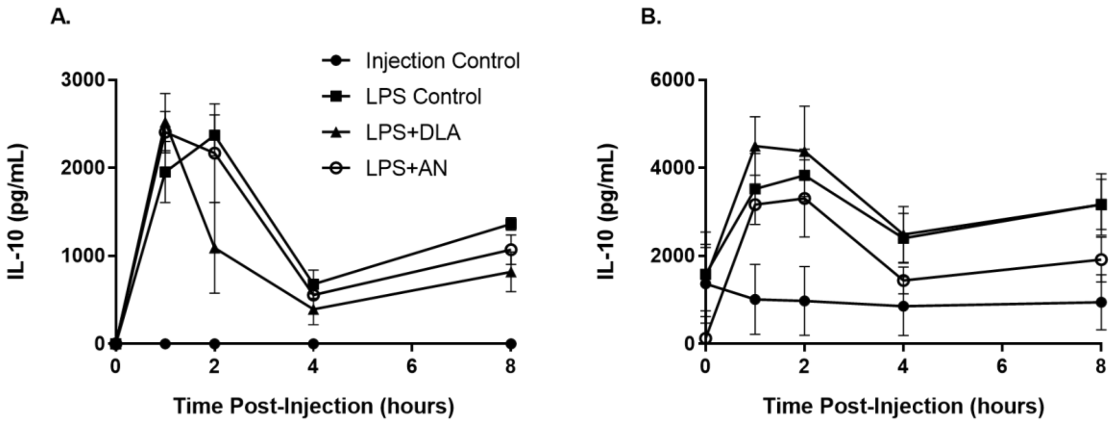

Figure A2. Production of anti-inflammatory cytokine IL-10 in male (**A**) and female (**B**) rats after LPS challenge and treatment with either pure artemisinin (AN) or DLA (equal artemisinin doses). $n = 5–6$ for experimental conditions, $n = 3$ for injection control; error bars = SEM.

Fecal Microbiome Analysis

We also examined the microbial populations present in fecal samples from male rats following administration of artemisinin or DLA to determine if either treatment affected the microbiome. Fecal samples from four DLA-treated and four artemisinin treated rats were collected at 1 and 8 h postgavage and stored at −80°C until analysis. Samples were sent on dry ice to the University of North Carolina Microbiome Core Facility (Chapel Hill, NC, USA) for 16S rRNA sequencing. The sequencing results showed that neither treatment altered the microbiome of the animals at either time point (Supplementary Table 1). These results suggested DLA and artemisinin would likely not alter the microbiome of patients, however, studies should be performed over a longer time course to confirm these results.

Table A1. Microbiome population by Phylum 1 h and 8 h after treatment with DLA or pure artemisinin in male rats.

	**Artemisinin Treated**		**DLA Treated**	
**Timepoint**	**1-hour**	**8-hour**	**1-hour**	**8-hour**
**Phylum**	**Average % Microbial Population ± SD**	**Average % Microbial Population ± SD**	**Average % Microbial Population ± SD**	**Average % Microbial Population ± SD**
**Unassigned**	0.23 ± 0.10%	0.15 ± 0.06%	0.15 ± 0.10%	0.18 ± 0.10%
**Actinobacteria**	0.35 ± 0.21%	0.80 ± 0.50%	0.28 ± 0.22%	0.58 ± 0.36%
**Bacteroidetes**	49.53 ± 9.38%	61.58 ± 7.62%	48.13 ± 6.78%	51.70 ± 8.58%
**Cyanobacteria**	0.13 ± 0.13%	0.38 ± 0.22%	0.00 ± 0.00%	0.45 ± 0.21%
**Deferribacteres**	0.03 ± 0.05%	0.00 ± 0.00%	0.00 ± 0.00%	0.00 ± 0.00%
**Firmicutes**	32.83 ± 8.99%	29.00 ± 8.89%	31.38 ± 17.17%	30.75 ± 20.26%
**Lentisphaerae**	0.00 ± 0.00%	0.00 ± 0.00%	0.00 ± 0.00%	0.00 ± 0.00%
**Proteobacteria**	3.85 ± 2.04%	2.28 ± 0.57	2.63 ± 1.99%	4.13 ± 1.82%
**Synergistetes**	0.00 ± 0.00%	0.00 ± 0.00%	0.00 ± 0.00%	0.00 ± 0.00%
**TM7**	0.33 ± 0.26%	0.30 ± 0.32%	0.63 ± 0.95%	0.23 ± 0.32%
**Tenericutes**	0.68 ± 0.57%	0.75 ± 1.17%	0.43 ± 0.17%	0.43 ± 0.10%
**Verrucomicrobia**	12.15 ± 10.64%	4.80 ± 6.54%	16.38 ± 10.95%	11.65 ± 11.70%

SD, Standard Deviation.
